# Curcumin-loaded PLGA-PEG nanoparticles conjugated with B6 peptide for potential use in Alzheimer’s disease

**DOI:** 10.1080/10717544.2018.1461955

**Published:** 2018-08-15

**Authors:** Shengnuo Fan, Yuqiu Zheng, Xuan Liu, Wenli Fang, Xiaoyu Chen, Wang Liao, Xiuna Jing, Ming Lei, Enxiang Tao, Qiulan Ma, Xingmei Zhang, Rui Guo, Jun Liu

**Affiliations:** a Department of Neurology, Sun Yat-sen Memorial Hospital, Sun Yat-sen University, Guangzhou, China;; b Key Laboratory of Biomaterials of Guangdong Higher Education Institutes Department of Biomedical Engineering, Jinan University, Guangzhou, China;; c Zhongshan City People’s Hospital, Zhongshan City, Guangdong Province, China;; d Department of Neurology, University of California, Los Angeles, CA, USA;; e Applied Immunology and Immunotherapy, Department of Clinical Neuroscience, Karolinska Institute, Center for Molecular Medicine, Karolinska University Hospital at Solna, Stockholm, Sweden;; f Laboratory of RNA and Major Diseases of Brain and Heart, Sun Yat-sen Memorial Hospital, Sun Yat-sen University, Guangzhou, China;; g Guangdong Province Key Laboratory of Brain Function and Disease, Zhongshan School of Medicine, Sun Yat-sen University, Guangzhou, China

**Keywords:** Curcumin, B6 peptide, nanoparticles, blood compatibility, Alzheimer’s disease

## Abstract

Alzheimer’s disease is a neurodegenerative disorder mainly characterized by β-amyloid deposit and tau hyperphosphorylation with no curative treatments. Curcumin (Cur) has been proved to have potential use in Alzheimer’s disease with its anti-amyloid, anti-inflammatory, and anti-oxidant properties, etc. However, its hydrophobicity and low bioavailability hinder its application. In this paper, we designed a novel brain-target nanoparticle, poly(lactide-co-glycolide)-block-poly(ethylene glycol) (PLGA-PEG) conjugated with B6 peptide and was loaded with Cur (PLGA-PEG-B6/Cur) and administered it into HT22 cells and APP/PS1 Al transgenic mice. The *in vitro* assays including dynamic light scattering (DLS), flow cytometry (FCM), red blood cell (RBC) lysis, and thromboelastography (TEG) analysis indicated that this nanoparticle could narrow the diameter of Cur, increase its cellular uptake and possess good blood compatibility. The results from Morris water maze proved that PLGA-PEG-B6/Cur could tremendously improve the spatial learning and memory capability of APP/PS1 mice, compared with native Cur. The *ex vivo* assays including Bielschowsky silver staining, immunostaining, and western blotting demonstrated that PLGA-PEG-B6/Cur could reduce hippocampal β-amyloid formation and deposit and tau hyperphosphorylation. Thus, we suggested that PLGA-PEG-B6/Cur nanoparticles would be of potential and promising use for the treatment of Alzheimer’s disease.

## 1. Introduction

Alzheimer’s disease (AD) is a progressive neurodegenerative disorder and the most prevalent cause of dementia, characterized by β-amyloid aggregation, tau hyperphosphorylation, neurofibrillary tangles, and neuron loss. According to the World Alzheimer Report 2015 (Prince et al., 2015), 46.8 million people worldwide are living with dementia and there will arise one new case every three seconds. However, currently there are only four effective drugs for the treatment of AD: the cholinesterase inhibitors donepezil, rivastigmine and galantamine, and the glutamate antagonist memantine (Scheltens et al., 2016). These drugs can only attenuate the symptoms of AD without curative effects. Therefore, new treatments that will prevent, delay or treat the symptoms of AD are in an urgent need.

Curcumin (Cur), a plant-derived polyphenolic compound, is naturally present in turmeric, a food and remedy widely used in India and China. Recent studies have illustrated that Cur possessed a variety of properties, such as anti-oxidant, anti-inflammation, anti-tumor, anti-viral, anti-bacterial, and had a potential against various disease, i.e. arthritis, allergies, diabetes, AD, and other chronic diseases (Perrone et al., 2015; Ganugula et al., 2017; Stanic, 2017). Cur targets the two histological markers of AD, amyloid beta (Aβ), and tau. It could decrease the production of β-amyloid, the formation and aggregation of senile plaque, tau hyperphosphorylation, and the formation of neurofibrillary tangles (Hamaguchi et al., 2010; Goozee et al., 2016; Tang & Taghibiglou, 2017). Additionally, it modulated other aspects of AD, for example, neuroinflammation, oxidative stress, cholesterol lowering, copper binding, microglial activity, insulin signaling pathway, and acetylcholinesterase inhibition (Goozee et al., 2016). Zheng et al. (2017) reported that Cur could ameliorate cognitive decline via inhibiting BACE1 expression, β-amyloid pathology, and synaptic degradation in 5 x FAD transgenic mice. In human neuroblastoma SH-SY5Y cells, Cur modulated PTEN/Akt/GSK-3β pathway and thus inhibited tau hyperphosphorylation (Huang et al., 2014). Cur administration inhibited the acetylcholinesterase activity via suppressing acetylcholinesterase gene expression in cadmium treated rats (Akinyemi et al., 2017). However, its poor bioavailability, which results from its low aqueous solubility and rapid metabolism and elimination from the body, and the poor permeability across the blood-brain barrier (BBB), constitute major hindrances for its application.

To overcome these limitations, many innovative technologies and drug delivery approaches are developed, such as isomerization, liposomes, polymeric nanoparticles, polymeric micelles, peptide/protein carriers, phospholipid complexes, cyclodextrins, solid dispersions, conjugates, and so on (Naksuriya et al., 2014). Poly (lactide-co-glycolide) (PLGA) and polyethylene glycol (PEG) are widely used for drug delivery purposes due to its biocompatibility and biodegradability (Jain, 2000; Fredenberg et al., 2011). Shaikh et al. (2009) reported that Cur-loaded PLGA nanoparticles could increase the bioavailability by at least nine-fold, compared with Cur alone . Studies from Khalil et al.(2013) illustrated that the PLGA and PLGA-PEG nanoparticles could increase the bioavailability of Cur by 15.6- and 55.4-fold, respectively by increasing the mean half-time of Cur, decreasing the metabolism of Cur and sustaining the Cur delivery, compared to the Cur aqueous suspension . Amin et al. (2017) administrated anthocyanins-loaded PLGA-PEG-2000 nanoparticles (An-NPs) in SH-SY5Y cell lines and the results indicated that compared to native bulk anthocyanins, An-NPs effectively attenuated Alzheimer’s markers (APP, BACE-1), inflammatory markers (NF-κB, TNF-α, iNOS), and apoptotic markers (Bax, Bcl_2_ and caspase-3).

The relative impermeability of the BBB resulting from the tight junctions between cerebral microvascular endothelial cells plays an important role in brain homeostasis, while it also greatly hinders drug delivery into the central nervous system (CNS) even incorporating them into nanoparticles (Li et al., 2011). In order to further improve the efficiency and specificity for brain delivery, receptors highly expressed on the endothelial cells, such as the insulin receptor, transferrin and integrin receptor, and the receptor-mediated transport (RMT) are of great interest to scientists (van Rooy et al., 2011). Among these various receptors, transferrin receptor (TfR) has been widely studied for BBB targeting over the past decade (Prades et al., 2012). However, it still presents some limitations, such as stability, synthesis procedure, and immunological response. B6 peptide (CGHKAKGPRK) is derived from a phage display library, targeting TfR as a substitute for transferrin protein and is considered as a promising candidate for drug delivery (Xia et al., 2000; Nie et al., 2011; Urnauer et al., 2017). Yin et al. (2015) synthesized sialic acid (SA)-modified selenium (Se) nanoparticles conjugated with B6 peptide (B6-SA-SeNPs), which showed high permeability across the BBB and thus effectively inhibited the aggregation of Aβ and disaggregated preformed Aβ fibrils. Liu et al. (2013) demonstrated that PEG-PLA-B6 encapsulated neuroprotective peptide nanoparticles exhibited higher accumulation in brain capillary endothelial cells and ameliorated learning impairment, cholinergic disruption, and neuron loss (Liu et al., 2013). All these above suggested that B6 peptide might be a potential tool for enhancing drug delivery into the CNS.

With these backgrounds, we designed a new nanomaterial, Cur-loaded PLGA-PEG-B6 micelles (PLGA-PEG-B6/Cur), with PLGA-PEG to increase the bioavailability and B6 peptide to enhance the BBB permeability of Cur. The aim of our study is to evaluate the potential efficacy of PLGA-PEG-B6/Cur for the treatment of AD. In our study, we would investigate the drug loading capacity, drug release kinetics, blood compatibility, cell viability, and cellular uptake of PLGA-PEG-B6/Cur *in vitro* and administrated it into APP/PS1 transgenic mice to evaluate its effect on cognitive impairment, Aβ, and tau pathologies by conducting Morris water maze, Bielschowsky silver staining, immunostaining, and western blotting.

## 2. Materials and methods

### 2.1. Materials

Dulbecco’s modified Eagle’s medium (DMEM), fetal bovine serum (FBS), phosphate buffer solution (PBS), neurobasal medium, N2 supplement, penicillin, and streptomycin were purchased from Gibco (New York, NY). A Cell Counting Kit-8 was obtained from Dojin Kagaku (Kumamoto, Kyushu, Japan). PEG 1500, D,L-Lactide, Glycolide were purchased from Daigang Biomaterial Co, Ltd (Jinan, China). Cur, stannous-2-ethylhexanote, acrylamide, dichloromethane, triethylamine, acryloyl chloride, diethyl ether, acetone, dimethyl sulfoxide (DMSO) and Calcium chloride (CaCl_2_) were gained from Aladdin Industrial Corporation (Shanghai, China). B6 peptide (CGHKAKGPRK) was supplied by GL Biochem Ltd (Shanghai, China). Blood from healthy volunteers was collected in sodium citrate tubes and the blood: anticoagulation ratio was 9:1. All other regents used were of analytical grade.

Antibody against Aβ_17-24_ was purchased from BioLegend (San Diego, CA). Antibodies against p-Thr231-Tau and p-Ser202-Tau were purchased from Thermo Fisher Scientific (Waltham, MA). Antibodies against p-Ser396-Tau, total Tau (Tau 46), Aβ (D54D2), BACE1, APP, Presenilin 1, actin, and horseradish peroxidase (HRP)-conjugated secondary antibody were acquired from Cell Signaling Technology (Danvers, MA). The chemiluminescent HRP substrate was obtained from Millipore (Billerica, MA). All other routine experiment reagents and supplies were supplied by Thermo Fisher, Invitrogen (Carlsbad, CA) and MR Biotech (Shanghai, China).

### 2.2. Synthesis of PLGA-PEG-B6

Synthesis of PLGA-PEG copolymers was done through ring-opening method. Briefly, PEG 1500 (6 g) was loaded to a stainless steel reactor and heated at 150 °C under 5 mmHg vacuum for 2 h in order to dry. Then D, L-lactide (9.0 g) and glycolide (3.0 g) were loaded to the reactor and heated at 150 °C under vacuum for 30 min. Stannous 2-ethylhexanoate (0.04 g) was added as catalyst and the heating was continued at 160 °C for 6 h under vacuum. When the reaction completed, the copolymers were dissolved in 4 °C cold water and then heated to 80 °C to precipitate and remove unreacted materials and water-soluble impurities. The purification procedure was done three times and purified PLGA-PEG copolymers were dried at 37 °C under vacuum to get constant weight.

The next step was to synthesize acrylated PLGA-PEG. PLGA-PEG (1 g) was sufficiently dissolved in dichloromethane (15 mL) and the solution was repeatedly vacuumized and purged with nitrogen. Triethylamine and acryloyl chloride was respectively dissolved in 5 mL dichloromethane and dripped into the PLGA-PEG solution in sequence in an ice bath and under dark condition. The molar ratio of PLGA-PEG, triethylamine, and acryloyl chloride was 1:8:8. The chemical reaction lasted for 24 h at room temperature. The solution was filtered to remove triethylamine hydrochloride crystal and the filtrate was precipitated in a ten-fold volume cold diethyl ether. The sediment was dried at 40 °C under vacuum, the resultant obtained was acrylated PLGA-PEG.

Then, acrylated PLGA-PEG and the B6 peptide were dissolved in phosphate buffered saline at a molar ratio of 1:1 with the adjusted pH value close to 7and reacted at room temperature for 24 h. Then the solution was dialyzed against deionized water and lyophilized. Through the above procedure, PLGA-PEG-B6 was obtained.

B6-functionalized PLGA-PEG were prepared via a maleimide-thiol coupling reaction. Briefly, acrylated PLGA-PEG and the B6 peptide were dissolved in phosphate buffered saline at a molar ratio of 1:1 with the adjusted pH value close to 7 and reacted at room temperature for 24 h. After the incubation period, the polymers were collected by centrifugation at 12,000 rpm for 15 min and washed twice with Milli-Q water. The PLGA-PEG-B6 was obtained and used for further analyses.

### 2.3. Characterizations of PLGA-PEG-B6

The ^1^H nuclear magnetic resonance (NMR) spectra were determined on an AV 3000 Supercon spectrometer (Bruker Optics, Ettlingen, Germany). Fourier transform infrared (FTIR) analysis was conducted using the attenuated total reflectance (ATR) technique (Bruker) and the spectra were recorded in the absorbance mode from 500 to 4000 cm^−1^. The particle sizes and zeta potentials were determined by dynamic light scattering (DLS) with a Mastersizer 2000 laser diffractometer (Malvern Instruments, Worcestershire, UK). Before the measurements, the nanoparticles were dissolved in 1 mL distilled water. Gel permeation chromatography (GPC) was performed in tetrahydrofuran (THF) with a Waters As- scope Model ALC/GPC 244 apparatus. THF was used as a mobile phase with a flow rate of 1.0 mL/min.

### 2.4. Drug loading capacity and cumulative release profiles

PLGA-PEG-B6/Cur micelle was prepared by solvent evaporation method. In brief, Cur and PLGA-PEG-B6 were dissolved in acetone. The suspension was stirred at high speed and dripped into deionized water. Once acetone evaporated completely, the suspension was filtered to eliminate the unloaded Cur. The filtrate was lyophilized to obtain the Cur-loaded PLGA-PEG-B6 (PLGA-PEG-B6/Cur) nanoparticles.

The loading capacity of Cur in the PLGA-PEG-B6/Cur nanoparticles were determined by ultraviolet spectrophotometer (Mathew et al., 2012). Firstly, the standard curve of Cur was plotted. In short, Cur (10 mg) was dissolved in DMSO and diluted to different concentrations (1, 5, 10, 12, 14, 16, 18, 20, 25, and 30 μg/mL). The absorbance values at 425 nm were measured to get the concentration-absorbance standard curve of Cur. To calculate the loading capacity of Cur, PLGA-PEG-B6/Cur powder was dissolved in DMSO and the absorbance value was measured at 425 nm. The loading capacity (LC) of Cur could be calculated using the following Equation (1):

(1) $$\text{LC}\;\left(\text{\%}\right)\text{=}\frac{\text{W}\text{eight}\;\text{of}\text{ Cur }\text{in}\;\text{nanoparticles}}{\text{Total}\;\text{nanoparticles}\;\text{weight}}\;\text{×}\;\text{100\%}$$

The release profiles of Cur from the nanoparticles were determined as described by Liu et al. (2013). In brief, PLGA-PEG-B6/Cur (10 mg) was diluted by 10 mL normal saline and the concentration of the solution was 1 mg/mL. The solution was transferred to a dialysis bag whose molecular weight cut off was 500. The dialysis bag was put into 10 mL PBS (0.01M, pH = 7.4) release medium and was placed on a shaking bed with a constant temperature of 37 °C to investigate the drug release profiles. At predetermined time intervals (0, 0.25, 0.5, 1, 2, 4, 6, 7, 8, 10, 12, 24, 48, and 72 h), 3 mL release fluid was taken out and fresh release medium of the same volume was supplemented. The absorbance values of the extracted release fluid were measured at 425 nm by ultraviolet spectrophotometer and the cumulative release of Cur were calculated accordingly. The data analyzed were the mean values from three independent experiments.

### 2.5. Reb blood cell (RBC) lysis

The RBC lysis of the PLGA-PEG-B6/Cur nanoparticles was measured as described by Liu et al. (2016). In short, RBC suspension (50 μL, 16% in PBS, v/v) was added into 1 mL PBS, which contained different concentrations of the nanoparticles. As positive control, 50 μL RBC suspension was added into 1 mL water to yield complete hemolysis. On the contrary, 50 μL RBC suspension was added to 1 mL PBS as a negative control. The mixtures were centrifuged at 1000 × g for 5 min and the supernatants were collected after incubation for a certain time. The absorbance values of the released hemoglobin (Hb) were measured at 540 nm with a microplate reader (SpectraMax M5, Sunnyvale, CA). The hemolysis ratio was calculated following the Equation (2):

(2) $$\text{Hemolysis}\;\left(\text{\%}\right)=\;\frac{\mathrm{As}\text{-}\mathrm{An}}{\mathrm{Ap}\text{-}\mathrm{An}}\;\text{×}\text{ 100\% }$$

where *A_s_, A_n_,* and *A_p_* are the absorbance values of the samples, negative, and positive controls. All data analyzed were from three independent experiments.

### 2.6. Thromboelastography (TEG)

Fresh citrate whole blood and different concentrations of the nanoparticles were mixed at a volume ratio of 9:1 in kaolin-containing tubes. Then 340 μL blood/nanoparticles mixture and 20 μL CaCl_2_ (0.2 M) solution were added into a TEG cup. The coagulation process was recorded at 37 °C by a Thromboelastograph Hemostasis System 5000 (Hemoscope Corporation, Niles, IL).

### 2.7. *In vitro* studies

#### 2.7.1. Cytotoxicity studies

The cytotoxicity of nanoparticles was evaluated in HT22 cells following the protocol of Cell Counting Kit-8 (CCK8) assays. Briefly, cells were cultured in 96-well plates at a density of 2 × 10^4^ cells/well in 100 μL of growth medium (DMEM +10% FBS +1% penicillin/streptomycin) at 37 °C incubator with 5% CO_2_ for 24 h and then differentiated in neurobasal medium with N2 supplement for another 24 h. After that, cells were respectively administrated with different concentrations (50, 100, 200, and 500 μg/mL) of the samples (PLGA-PEG-B6 nanoparticles, native Cur, Cur-loaded PLGA-PEG nanoparticles, and PLGA-PEG-B6/Cur nanoparticles). Cell control group was treated with equivalent volume of fresh medium and blank group was only treated with medium (without cells). After 24 h, 10 μL CCK8 was added into the medium and the absorbance values were detected at 450 nm with a multifunctional microplate reader (SpectraMax M5, Sunnyvale, CA) after 2 h. Untreated cells were taken as positive controls with 100% cell viability. Each measurement was conducted in triplicates and the data analyzed were the mean values of three different experiments.

#### 2.7.2. Cellular uptake studies

Flow cytometry (FCM) was used for quantitative cellular uptake study. HT22 cells were seeded in 12-well plates of 2 × 10^5^ cells/well, kept at 37 °C incubator for 24 h and then differentiated for another 24 h. In order to investigate the cellular uptake efficiencies of PLGA-PEG-B6/Cur nanoparticles in HT22 cells of different doses and incubation time, several concentrations (50, 100, 200, and 500 μg/mL) of particles were added into the culture medium and incubated for different time (2, 4, 6, 8, and 12 h), respectively. Besides, the cellular uptake efficiencies of native Cur and Cur-loaded PLGA-PEG nanoparticles (PLGA-PEG/Cur) were also evaluated. Since Cur or Cur-loaded nanoparticles are auto-fluorescent in fluorescein isothiocyanate (FITC) channel, no other cell staining technique was used. Cells without treatment were considered as negative controls. The percentages of fluorescence positive cells and the fluorescence intensity were compared among different groups and data analyzed were from three independent experiments.

### 2.8. *In vivo* studies

#### 2.8.1. Animals and drug administration

APPswe/PS1dE9 double transgenic mice (APP/PS1 mice) were purchased from the Model Animal Research Center of Nanjing University (Nanjing, China; strain type B6C3-Tg [APPswe, PSEN1dE9] 85Dbo/J). All the mice were kept in a room (22 ± 2 °C) maintained on a dark-light light cycle of 12 h with free access to food and water. In this study, 32 SPF Nine-month-old male APP/PS1 double transgenic mice (Tg, 28.30 ± 3.64 g) were used, eight age-matched male wild-type littermates (WT) were used as controls. The mice were randomly divided into five groups: wild type (WT, *n* = 8), transgenic control (APP/PS1, *n* = 8), transgenic Cur (*n* = 8), transgenic PLGA-PEG/Cur (NT, *n* = 8), and transgenic PLGA-PEG-B6/Cur (Tar, *n* = 8).

APP/PS1 transgenic mice were intraperitoneally injected with Cur, PLGA-PEG/Cur nanoparticles, PLGA-PEG-B6/Cur nanoparticles (all these three groups with an effective concentration of 25 mg/kg/d, *n* = 8). APP/PS1 mice (*n* = 8) and WT mice (*n* = 8) were treated with the same volume of 0.9% normal saline, served as positive and negative control groups, respectively. The described treatments were performed weekly, 12 times in total. All experimental procedures strictly followed the regulations of the Institutional Animal Care and Use Committee (IACUC) of Sun Yat-sen University, Guangzhou, China.

#### 2.8.2. Morris water maze (MWM)

The Morris water maze (MWM) is a test to force mice to swim and search for the platform hidden beneath the water, aiming to evaluate the spatial learning and memory ability. We followed the procedures as described (Xiao et al., 2016; Chen et al., 2017). The MWM test consists of a five-day orientation navigation experiment and a one-day space exploration experiment. Before the test, all the mice were put into the pool to swim for 2 min and then kept on the platform for 20 s to get accustomed to the environment. The timer set for the navigation experiment was 90 s. The mice were released from four of the quadrants, respectively. Once the mice reached the platform and stayed there for 3 s within 90 s, the timer would automatically stop and record the time as the escape latency. The mice would be forced to stay on the platform for 20 s if they could not find the platform within the scheduled time. The mice were trained four times a day and the data obtained were the mean values of the escape latencies of the four quadrants. After each trail, the mice were dried off in their housing facilities. On the sixth day in the space exploration experiment, the platform was removed and the mice were allowed to swim for 60 s freely. The percentage of time spent in the target quadrant (where the platform was previously located) and the numbers crossing the platform were recorded by a video tracking equipment and processed by a computer equipped with an analysis-management system.

#### 2.8.3. Tissue preparation

After the behavioral tests, the mice were sacrificed under deep anesthesia with 10% chloral hydrate. For Bielschowsky silver staining, immunohistochemical (IHC), and immunofluorescence (IF) staining, the mice were perfused transcardially with normal saline and 4% paraformaldehyde in sequence and then the removed brains were post-fixed in 4% paraformaldehyde (PFA) overnight, dehydrated in 30% sucrose in phosphate buffer (0.1 M) for 48 h and finally cut into a thickness of 15 μm each slice coronally. For Western blotting, mice were perfused with normal saline and the removed brains were immediately frozen at −80 °C.

#### 2.8.4. Bielschowsky silver staining and immunostaining

Bielschowsky silver staining, IHC and IF staining were performed according to the previously published protocols (Schwab et al., 2004; Zhang et al., 2014; Fan et al., 2015). In brief, brain sections of hippocampus, mainly Cornu Ammonis 1 (CA)1, were fixed with 10% glacial acetic acid, washed with double steamed water and then stained with Bielschowsky silver staining reagent. For IF/Aβ, sections of hippocampus were blocked with goat serum, incubated with anti-Aβ (D54D2; 1:500) primary antibody overnight at 4 °C and then fluorescent secondary antibody. For IHC/Aβ, sections of hippocampus were incubated with anti-Aβ 17-24 (1:500) antibody overnight at 4 °C, followed by second biotinylated goat anti-mouse IgG antibody at room temperature and finally with HRP-conjugated streptavidin. Images were acquired using a Zeiss LSM 710 laser confocal scanning microscope (Zeiss, Germany). The analysis of Aβ burden was expressed as the relative density normalized to APP/PS1 group.

#### 2.8.5. Western blotting

For the Western blotting assay, mouse brains were homogenized in ice-cold lysis (50 mmol/L Tris (pH =7.4), 150 mmol/L NaCl, 1% Triton X-100, 1% sodium deoxycholate, 0.1% sodium dodecyl sulfate (SDS)) according to the protocol. The sample proteins of mouse hippocampus were electrophoretically separated using 10% bicine/tris gel and transferred to polyvinylidene difluoride membranes. After blocking in 5% (w/v) nonfat milk, the membranes were hybridized overnight in 4 °C with appropriate primary antibodies (p-Ser396-Tau 1:1000, p-Ser202-Tau 1:1000, p-Thr231-Tau 1:1000, total Tau (Tau 46) 1:1000, Aβ 1:1000, BACE1 1:1000, APP 1:1000, PS1 1:1000, and Actin 1:2000), washed and then incubated for 1 h with the appropriate HRP-conjugated secondary antibody in 5% (w/v) nonfat milk. Blots were developed into the films and images were scanned afterwards. Densitometric analysis was performed using Image J software (NIH, Bethesda, MD).

### 2.9. Statistical analysis

All data were expressed as mean ± standard deviation (SD) and the differences among groups were analyzed with one-way analysis of variance (ANOVA) followed by a Bonferroni *post-hoc* test using the SPSS 20.0 software (SPSS Inc., Chicago, IL). *p <* .05 was considered statistically significant.

## 3. Results

### 3.1. Synthesis and characterizations of nanoparticles

**Figure 1.**
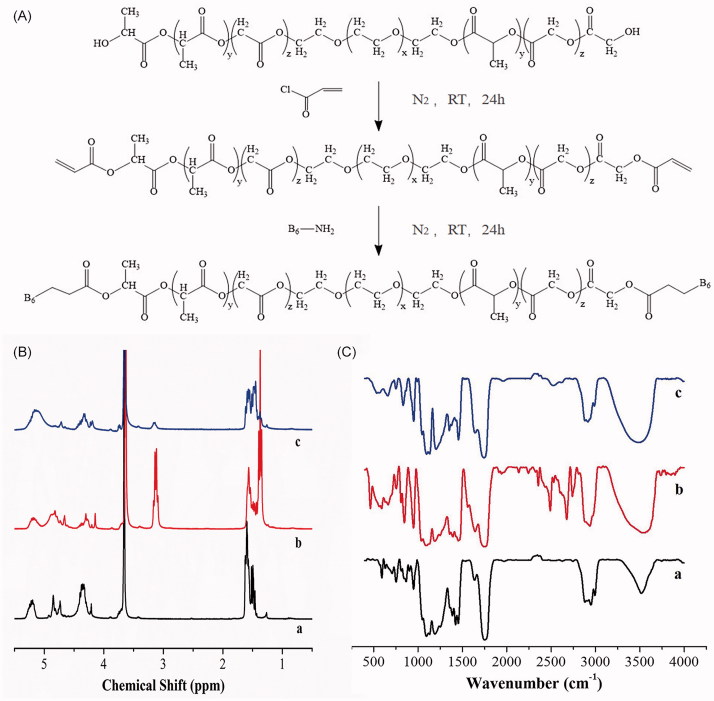
(A) General procedure for the synthesis of PLGA-PEG-B6 nanoparticles. (B) ^1^H NMR spectra of (a) PLGA-PEG, (b) acrylated PLGA-PEG, and (c) PLGA-PEG-B6. (C) FTIR spectra of (a) PLGA-PEG, (b) acrylated PLGA-PEG, and (c) PLGA-PEG-B6.

The synthesis scheme of PLGA-PEG-B6 is shown in Figure 1(A) and the formation of PLGA-PEG-B6 was confirmed via ^1^H NMR spectra and FTIR, as shown in Figure 1(B,C).

**Figure 2.**
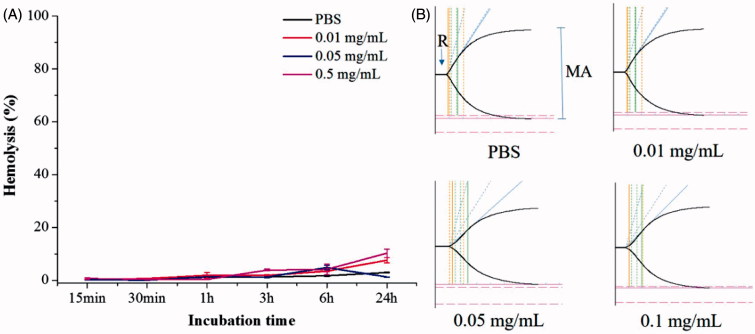
RBC lysis and TEG of the PLGA-PEG-B6/Cur nanoparticles. (A) Effect of different concentrations of PLGA-PEG-B6/Cur nanoparticles on RBC hemolysis. (B) Effect of PLGA-PEG-B6/Cur nanoparticles on blood coagulation. Data represented as mean ± SD (*n* = 3).

The ^1^H NMR spectra of PLGA-PEG, acrylated PLGA-PEG, and PLGA-PEG-B6 are shown in Figure 1(B). In Figure 2(A) (a), the signals pertaining to PLGA-PEG are found in δ = 5.19 ppm (CH of lactide), 1.52 ppm (CH_3_ of lactide), 4.7 ppm (CH_2_ of glycolide), and 4.30 ppm (CH_2_ of ethylene glycol). In Figure 2(A) (b), new peaks at 4.5 to 5 ppm were attributed to the protons of -CH = CH_2_, indicating the successful synthesis of acrylated PLGA-PEG. In Figure 1(B) (c), the peaks at 4.5 to 5 ppm disappeared, showing that the reaction occurred on the -CH = CH_2_ group.

The FTIR spectra of PLGA-PEG, acrylated PLGA-PEG, and PLGA-PEG-B6 are shown in Figure 1(C). The peak at 3522 cm^−1^ is the broad O-H stretching peak. In Figure 1(C) (b), the intensity of the peak at 1636 cm^−1^ was strengthened, which was attributed to the overlapping of C = C stretching peak, indicating that the successful synthesis of acrylated PLGA-PEG. In Figure 1(C) (c), the peak at 1636 cm^−1^ narrowed down, indicating that the reaction occurred with the C = C groups. At the same time, the peak occurred at 2520 cm^−1^ was attributed to the -SH stretching peak of the B6 peptide. The FTIR results and the ^1^H NMR results indicated that B6 peptide was successfully grafted on the backbone of the PLGA-PEG polymers.

As can be seen from the result (Figure S1), both PLGA-PEG-B6 and PLGA-PEG-B6/Cur nanoparticles were round under dynamic light scattering (DLS). The sizes of the PLGA-PEG-B6 nanoparticles were all less than 100 nm. The encapsulation of Cur slightly increased the sizes, but most of them were less than 150 nm, ranging from 50 to 250 nm. The zeta potential of PLGA-PEG-B6 was 3.83 ± 0.89 mV.

The number-average molecular weight (Mn) and weight-average molecular weight (Mw) of PLGA-PEG were 12,000 and 2500, respectively (Supplementary Table S1). The polydispersity( Pd = Mw/Mn) of copolymer was about 2.08, which showed a symmetric peak and had a relative narrow molecular weight. Unimodal GPC with low polydispersity confirmed the formation of PLGA-PEG copolymer. It is of interest to note that the Mw of acrylated PLGA-PEG, PLGA-PEG-B6, and PLGA-PEG-B6/Cur are 25,600, 26,000 and 26,600, respectively, which demonstrate the synthesis of acrylated PLGA-PEG, PLGA-PEG-B6, and PLGA-PEG-B6/Cur are successful.

### 3.2. Drug loading capacity and release profiles

According to the plotted standard curve of Cur, the loading capacity of Cur in PLGA-PEG-B6/Cur nanoparticles was 15.6%.

The time-dependent drug release profiles of Cur from micelles were investigated at pH 7.4, which was equivalent to the pH value of human blood. As shown in Supplementary Figure S2, PLGA-PEG-B6/Cur nanoparticles exhibited a biphasic release pattern characterized by an initial release of 29.60 ± 0.70% Cur from the PLGA-PEG-B6/Cur nanoparticles within the first hour, followed by a sustained release to 78.01 ± 1.55% over 72 h.

### 3.3. Blood compatibility

Good blood compatibility is the foundation of drug utilization, which can reduce the change of its structure or function. Hemolysis rate and thromboelastography are usually used to evaluate blood compatibility. Hemolysis refers to the damage of cell membrane integrity and subsequently the release of hemoglobin from RBCs, which reflects the interaction of biomaterials with RBC membrane. The effects of different concentrations of PLGA-PEG-B6/Cur nanoparticles on RBC hemolysis are shown in Figure 2(A). The hemolysis percentages between normal saline and nanoparticles solution was of no significant difference, even when the concentration reached up to 0.5 mg/mL, indicating that the nanoparticles possessed no obvious hemolytic reaction.

TEG is widely used to detect the whole blood coagulation process clinically. The TEG assay has four main parameters: (1) reaction time (*R*), the time from the CaCl_2_ initiator administration to the formation of the initial fibrin; (2) coagulation time (*K*), the time of the clot formation; (3) α angle, the rate of clot polymerization or fibrin crossing; (4) maximum amplitude (MA) of the tracing, the maximum clot strength. TEG traces and the corresponding parameters of *R*, *K*, α angle, and maximum amplitude (MA) are shown in Figure 2(B) and Supplementary Table S2. The nanoparticles of different concentrations displayed a similar shape to the normal saline and the *R* of nanoparticles were all in normal range. The lower MA values implied that the platelet activities were lower. The increase in the *K* value and the decrease in the α angle indicated the reduction of fibrinogen activities. In conclusion, these results implied that the PLGA-PEG-B6/Cur nanoparticles with a concentration less than 0.05 mg/mL had little influence on the clotting system in the whole blood. 

### *3.4. In vitro* results

#### 3.4.1. Cytotoxicity studies

**Figure 3.**
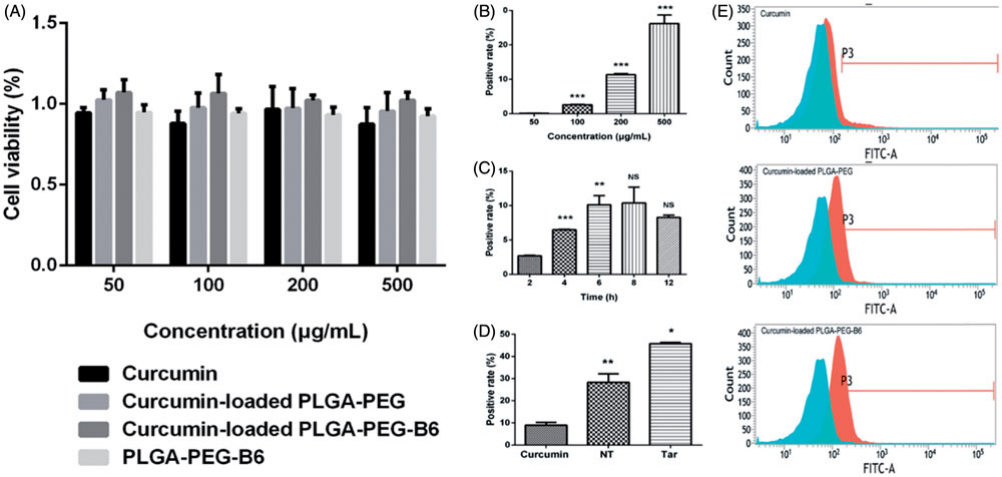
Cell viabilities and cellular uptake efficiency. (A) Results of cell viabilities by CCK-8 assay after 24 h incubation with different concentrations (50, 100, 200, and 500 µg/mL) of Cur, Cur-loaded PLGA-PEG, Cur-loaded PLGA-PEG-B6, and PLGA-PEG-B6, respectively. Cells without treatment were regarded as the control group. Data as mean ± SD, *n* = 3. HT22 cellular uptake (B) 50–500 μg/mL Cur-loaded PLGA-PEG-B6 nanoparticles at 37 °C incubation for 4 h. (C) 200 μg/mL Cur-loaded PLGA-PEG-B6 nanoparticles at 37 °C incubation for different times. (D) 500 μg/mL Cur, Cur-loaded PLGA-PEG and Cur-loaded PLGA-PEG-B6 nanoparticles at 37 °C incubation for 6 h, respectively. (E) Flow cytometry for Cur, Cur-loaded PLGA-PEG and Cur-loaded PLGA-PEG-B6 nanoparticles uptake (500 μg/mL, 6 h) in HT22 cells. Data as mean ± SD, *n* = 3. *p < .05, ** *p* < .01,***p ≤ .001, and no significant difference (NS), versus the former group.

Cytotoxicity of Cur, PLGA-PEG/Cur nanoparticles, PLGA-PEG-B6/Cur nanoparticles, and PLGA-PEG-B6 nanoparticles were investigated in HT22 cells after 24 h incubation by the CCK-8 assay (Figure 3(A)). We proved that all the particles did not affect cell viabilities within the concentration of 500 μg/mL, which was a relatively high concentration. It could be concluded that native Cur, PLGA-PEG/Cur, and PLGA-PEG-B6/Cur nanoparticles were biocompatible and possessed relatively low toxicity profiles.

#### 3.4.2. Cellular uptake studies

In this study, we conducted flow cytometry to measure the cellular uptake in HT22 cells in FITC channel. As shown in Figure 3(B–E), PLGA-PEG-B6/Cur nanoparticles tremendously increased its cellular uptake in HT22 cells, compared with native Cur (Figure 3(D, E)). The uptake efficiency rose from 8.94 ± 1.10 to 45.72 ± 0.48% (Figure 3(D)). Besides, the cellular uptake of PLGA-PEG-B6/Cur nanoparticles was dose-dependent and time-related (Figure 3(B,C)). 

### *3.5. In vivo* results

#### 3.5.1. PLGA-PEG-B6/Cur improved the spatial learning and memory capability of APP/PS1 mice in the MWM

**Figure 4.**
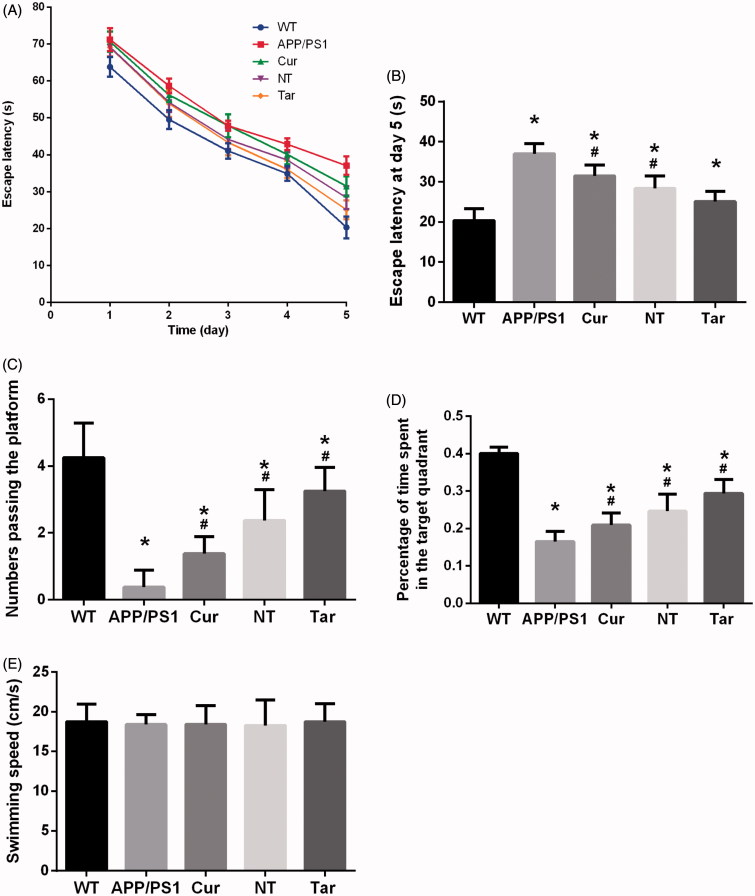
Cur-loaded PLGA-PEG-B6 nanoparticles attenuated the learning and memory impairment of APP/PS1 mice. (A) Comparison of escape latency of each group from day 1 to day 5 in learning trials. (B) Comparison of escape latency of each group at day 5. PLGA-PEG-B6/Cur nanoparticles (25.113 ± 2.506) shortened the time finding the platform than the APP/PS1 group (37.034 ± 2.497). (C) Numbers passing the platform within 60 s in the probe trial. PLGA-PEG-B6/Cur nanoparticles (3.250 ± 0.707) greatly increased the numbers passing the platform than the APP/PS1 group (0.375 ± 0.518). (D) Percentage of time spent in the target quadrant in the probe trial. PLGA-PEG-B6/Cur nanoparticles (0.294 ± 0.037) spent more time in the target quadrant searching for the platform than the APP/PS1 group (0.165 ± 0.027). (E) Comparison of swimming speed of each group. There was no significant difference among groups in swimming speed. (*n* = 8, *p < .05 versus WT group, ^#^*p* < .05 versus former group). WT: wild type; APP/PS1: saline; Cur: curcumin; NT: PLGA- PEG/Cur nanoparticles; Tar: PLGA-PEG-B6/Cur nanoparticles.

To investigate the effect of PLGA-PEG-B6/Cur nanoparticles on the spatial learning and memory capability, we performed the Morris water maze test. In the orientation navigation experiment from the first to the fifth day, we analyzed the escape latencies of the mice and found that they spent less time finding the platform as time passed by and PLGA-PEG-B6/Cur nanoparticles shortened the escape latency than the APP/PS1 group (Figure 4(A, B)). The mice pretreated with PLGA-PEG-B6/Cur nanoparticles showed more times crossing the platform and spent more time searching the platform in the target quadrant, when compared with the APP/PS1 and Cur group (Figure 4(C,D)). The swimming speeds among these groups were of no difference, which could exclude the physical influence on the cognition test (Figure 4(E)). The APP/PS1 mice is a good model for Alzheimer’s disease research and the spatial learning ability is deteriorated. However, PLGA-PEG-B6/Cur nanoparticles could remarkably improve the cognitive impairment in APP/PS1 mice, which would be of potential use in Alzheimer’s disease.

#### 3.5.2. PLGA-PEG-B6/Cur reduced hippocampal Aβ burden in APP/PS1 mice

**Figure 5.**
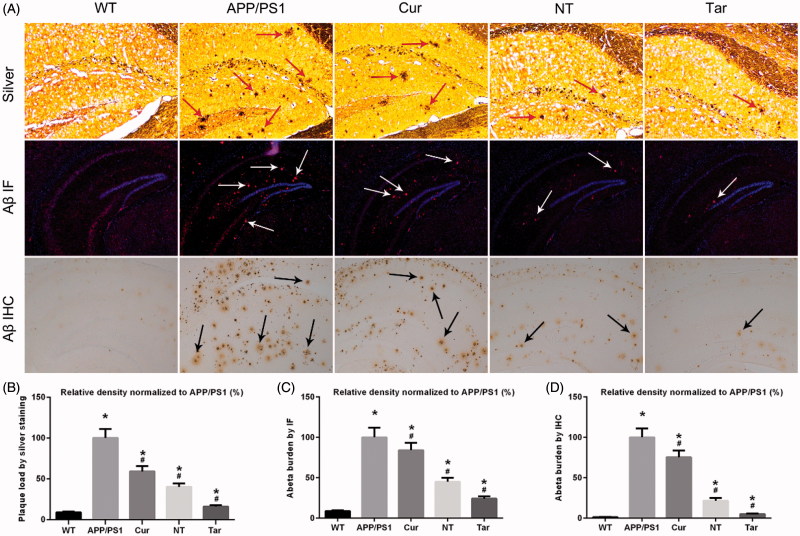
Cur-loaded PLGA-PEG-B6 nanoparticles reduced hippocampal Aβ burden in APP/PS1 mice. APP/PS1 mice exerted mass amyloid formation (red, white and black arrows) in the hippocampal area. Cur and NT moderately reduced Aβ plaques formation when compared with APP/PS1 control group. Most notably, Tar significantly attenuated this pathology. (A) Silver staining (red arrows), immunofluorescence (IF, white arrows), and immunohistochemistry (IHC, black arrows) of Aβ plaques of each group. (B–D) Comparison of relative density of Aβ plaque in each group by silver staining, Aβ IF, and Aβ IHC, respectively. APP/PS1 mice and WT mice treated with saline served as positive and negative control. (*p < .05 versus WT group, ^#^*p* < .05 versus former group).

Given the previous reports showing that Cur decreased AD-like pathology in APP/PS1 mice (Huang et al., 2012; Feng et al., 2016), we aimed to investigate whether PLGA-PEG-B6/Cur nanoparticles could further attenuate AD-related Aβ plaques formation in AD model mice. APP/PS1 mice were treated with saline (APP/PS1), Cur, PLGA-PEG/Cur nanoparticles (NT), PLGA-PEG-B6/Cur nanoparticles (Tar), followed by Bielschowsky silver staining and IF/IHC of Aβ in brain sections. As shown in Figure 5, when compared with the WT mice, APP/PS1 mice exerted marked cerebral Aβ plaques deposition. While the Cur g and NT group showed moderate reducing effects, Tar group manifested the most prominent reduction in this pathology. These results suggested that PLGA-PEG-B6/Cur nanoparticles might be effective in treating AD by targeting Aβ pathology.

#### 3.5.3. PLGA-PEG-B6/Cur reduced hippocampal Aβ production and decreased tau phosphorylation in APP/PS1 mice

**Figure 6.**
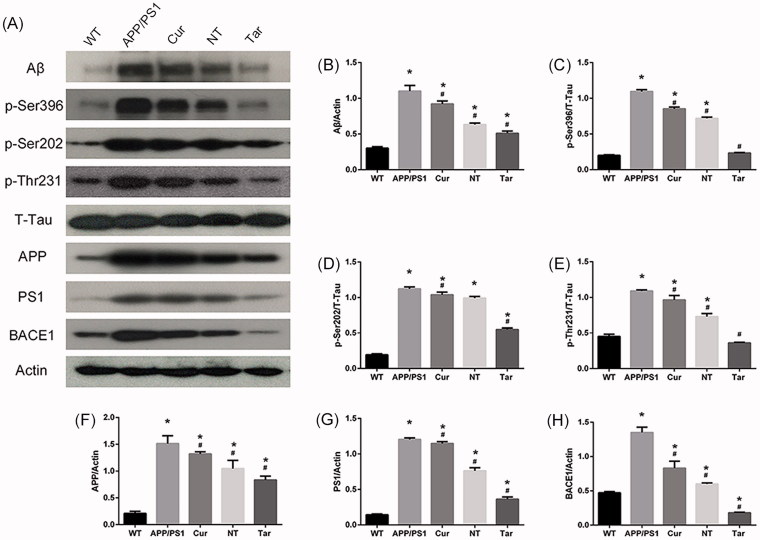
PLGA-PEG-B6/Cur nanoparticles reduced hippocampal Aβ production and decreased tau phosphorylation in APP/PS1 mice. (A) Protein bands of Aβ, p-Tau (Ser396, Ser202 and Thr231), T-Tau, APP, PS1, BACE1, and actin of each group, respectively. Actin served as the internal control. (B–H) Comparison of the protein expression of Aβ, p-Tau (Ser396, Ser202 and Thr231), APP, PS1, and BACE1 among these five groups. Densitometry analysis data showed that PLGA-PEG-B6/Cur nanoparticles (Tar) significantly decreased the band density ratios of Aβ, p-Tau, BACE1, APP, and PS1 to actin/T-tau. *p < .05 versus WT group, ^#^*p* < .05 versus former group.

Since the Bielschowsky silver staining and immunostaining results suggested that PLGA-PEG-B6/Cur nanoparticles potentially reduced AD pathology in APP/PS1 mice, we further evaluated the Aβ-related proteins and p-tau protein level in mouse brain. As shown in Figure 6, the protein level of Aβ and the phosphorylation level of tau were significantly more in APP/PS1 mice than in WT mice. However, Cur and NT could somehow decrease these expressions, while Tar showed the most outstanding inhibitory effect. Interestingly, inhibition of BACE1, APP, and PS1 could also be found, suggesting that APP processing and γ- secretase cleavage might be altered by PLGA-PEG-B6/Cur nanoparticles in APP/PS1 mice.

## 4. Discussion

Although the current hypotheses emphasize different stories, including β-amyloid deposit, tauopathy, oxidative stress (OS), calcium overload, cholinergic, and glutamatergic neurotransmission alterations, β-amyloid cascade and tauopathy are still the most widely accepted central factors triggering and/or accelerating the AD pathogenesis (Roberson et al., 2007; Jucker & Walker, 2015; Sanabria-Castro et al., 2017). It is believed that an imbalance between production and clearance of β-amyloid is the triggering event and is the key factor responsible for other abnormalities observed in AD (Cummings et al., 2007; Yan, 2017), while tau protein arises as a secondary pathogenic event that subsequently worsens neurodegeneration (Roberson et al., 2007). Oddo et al. (2003) found that tauopathy was detected in the CA1 area at 15 months old 3 x Tg-AD mouse model. Over-deposited Aβ and hyperphosphorylated tau resulted in neurological deficits in synapses and neuronal circuits, both morphologically and functionally (Selkoe, 2008; Arendt, 2009). In line with these clinical researches and some other *in vivo* studies (Davis et al., 2017; Jin et al., 2017), our study also showed mass Aβ accumulation and phosphorylation of tau in APP/PS1 mouse brain.

The last few decades have witnessed phenomenal progress in understanding the pathogenesis of AD, however, clinical therapeutic strategies achieve negligible effect. Thus, novel approach that elicits both robust anti-β amyloidosis and anti-tauopathy activities may provide new solution to the future treatment for AD. Many natural bioactive compounds have come to the attention of researchers over the years, among which Cur is of great interest. Besides vast functions, such as anti-oxidant, anti-inflammation, anti-tumor et al., Cur also exerts properties in high Aβ affinity, anti-β amyloidosis, and anti-tauopathy in AD (Hamaguchi et al., 2010; Ngo & Li, 2012; Goozee et al., 2016; Tang & Taghibiglou, 2017). In agreement with the results of previous studies, the present study also revealed that native Cur could partially rescue memory loss and attenuate β amyloidosis and tau phosphorylation in APP/PS1 mice. However, native Cur did not obtain potent effect as expected, which might result from its poor bioavailability and low permeability across the BBB when administered peripherally.

To ameliorate the poor bioavailability and low permeability through the BBB and fully gain the beneficial properties from Cur, more and more researches were conducted upon nanoparticle systems, among which PLGA and PEG were proved to be outstanding in bio-safety and efficient in bioavailability (Jain, 2000; Fredenberg et al., 2011; Khalil et al., 2013; Paka & Ramassamy, 2017). In addition to bioavailability, BBB permeability remains to be another challenge of drug delivery system. As a dynamic structure between the blood and the brain, BBB is mainly formed by a group of endothelial cells, astrocytes, and the tight conjunctions between them, which makes restricted passage for certain substances to the brain (Pandey et al., 2016). According to the research of Visser et al. (2004), transferrin receptor (TfR) is highly expressed in brain capillary endothelia cells, and that is involved in the receptor-mediated transport (RMT) through the BBB to deliver the iron into the brain. This finding makes TfR possible to be a potential target for drug delivery system. However, the usage of large proteins, for example, transferrin protein or TfR antibodies, resulted in problems such as synthesis procedure, stability, and immunological response. Interestingly, B6 peptide, targeting TfR that presents in the brain capillary endothelial cells and in neurons, exerts high permeability across the BBB when conjugated with some nanoparticles (Liu et al., 2013; Yin et al., 2015). To acquire excellent bioavailability and permeability, PLGA-PEG-B6 nanoparticles loaded with Cur were developed in this study.

From DLS measurements, we find that PLGA-PEG-B6 nanoparticles were well accommodated in solution with an average diameter of less than 50 nm. Its mean size increased to around 100 nm after B6 conjugation, with a favorable size distribution. The nanoparticles synthesized revealed a low zeta potential of PLGA-PEG-B6 at 3.83 ± 0.89 mV and size of PLGA-PEG-B6/Cur nanoparticles around 100 nm, which were important because particles under 200 nm might be able to get through the BBB without much difficulty (Jain, 2012). The loading capacities of most nanomaterials were less than 10% in other researches (Anitha et al., 2011; Song et al., 2011; Liu et al., 2012; Zhao et al., 2012), while our study revealed a loading capacity of 15.6%, which was relatively high. Furthermore, the results of drug release profiles showed that PLGA-PEG-B6/Cur nanoparticles exhibited good sustained release, with only 29.60 ± 0.70, 47.50 ± 0.71, 66.16 ± 1.26, and 72.99% ± 4.07%, respectively of Cur released from the PLGA-PEG-B6/Cur nanoparticles at 1, 4, 8, and 24 h. The sustained release kinetics of Cur from micelles was mostly because Cur was incorporated inside the nanoparticle bilayers, which could greatly reduce its burst release and retard its release rate in blood plasm and thus increase its concentration in the central nervous system accordingly (Manju & Sreenivasan, 2011; Chen et al., 2015). Blood compatibility test revealed that PLGA-PEG-B6/Cur nanoparticles had little influence on the hemolysis and clotting systems, which implied that PLGA-PEG-B6/Cur nanoparticles were biologically safe and stable and might be an underlying effective drug for *in vitro* and/or *in vivo* studies.

To investigate the bio-safety and uptake property of the PLGA-PEG-B6/Cur nanoparticles, *in vitro* studies were conducted in HT22 cells. Cell viability analysis manifesting no significant difference in cytotoxicity could be observed in PLGA-PEG-B6/Cur nanoparticles and PLGA-PEG-B6 nanoparticles within 500 μg/mL, which was a relatively high concentration. Compared with native Cur, PLGA-PEG-B6/Cur nanoparticles tremendously increased its cellular uptake, with a dose- and time-dependent manner. Thus, PLGA-PEG-B6/Cur nanoparticles were regarded as a relatively safe and promising candidate for *in vitro* study.

As a popular rodent model of AD, APP/PS1 mice develop amyloid plaques and behavioral deficits around 6–7 months, presenting an age-dependent manner of Aβ deposition in senile plaques (Jankowsky et al., 2004). APP/PS1 mice have been extensively used to better understand the pathogenic mechanisms underlying synaptic dysfunction and memory impairment in AD, and to screen new therapeutic approaches (Gong et al., 2006; Perez-Gonzalez et al., 2013; Jiao et al., 2015; Jin et al., 2017). Our previous study also illustrated the characteristic histological lesions in this mouse model at the age of six months and once again proved it to be a good model (Xiao et al., 2016). Thus, in the present study, we used APP/PS1 mice to investigate the effect of PLGA-PEG-B6/Cur nanoparticles on the spatial learning and memory capability. After treated with different agents for three months, the Tar group crossed the platform most and spent most time searching the platform in the target quadrant, implying that PLGA-PEG-B6/Cur nanoparticles had the most excellent effect on cognitive impairment.

Aβ is the main component of brain senile plaques (Puzzo et al., 2015), resulting from the sequential cleavage of APP by two distinct enzymes: the BACE1 and the γ-secretase (Guglielmotto et al., 2011). The intraneuronal neurofibrillary tangles (NFT) caused by tau hyperphosphorylation is another hallmark of AD (Bloom, 2014). In the present study, we further performed silver staining and IF/IHC of Aβ in hippocampal CA1 area of each group. As a result, Cur and NT groups partially reduced Aβ plaques formation, while Tar group significantly attenuated amyloid burden. More interestingly, the western blotting results revealed that both Cur and NT could partially reduce the Aβ level and the phosphorylation level of tau in APP/PS1 mouse brain, while Tar demonstrated the most outstanding inhibitory effect. Along with reduction of Aβ expression and tau phosphorylation, inhibition of BACE1, APP, and PS1 could also be seen in this process. Our results suggested that PLGA-PEG-B6/Cur nanoparticles could improve learning and memory function and ameliorate AD pathologies by reducing tau phosphorylation and Aβ deposition, in which alteration of APP and β-secretase cleavage processing might be involved.

## 5. Conclusion

Our study explored the potential application of a novel nanomaterial, PLGA-PEG-B6/Cur nanoparticles, in attenuating memory loss, Aβ burden and tauopathy in APP/PS1 mice. The results of the study suggest that PLGA-PEG-B6/Cur nanoparticles possess promising properties in relieving β-amyloidosis and tauopathy, with good bio-safety and high bioavailability. These findings together indicate that PLGA-PEG-B6 can profoundly improve the delivery efficiency of nanoparticles to the brain and that PLGA-PEG-B6/Cur nanoparticles may serve as a promising therapeutic strategy for the treatment of AD in the future.

## Supplementary Material

Supplementary_Data-0402.docx
