# Supplementary material for: Curcumin-loaded PLGA-PEG nanoparticles conjugated with B6 peptide for potential use in Alzheimer’s disease
Source: Drug Deliv. 2018 Aug 15;25(1):1091–102. doi: 10.1080/10717544.2018.1461955 (PMC6116673; doi:10.1080/10717544.2018.1461955)
Supplement: Supplementary_Data-0402.docx [file IDRD_A_1461955_SM7575.docx]

**Curcumin-Loaded PLGA-PEG Nanoparticles conjugated with B6 Peptide for Potential Use in Alzheimer’s disease**

Shengnuo Fan ^a, 1^, Yuqiu Zheng ^a, 1^, Xuan Liu ^b^, Wenli Fang ^a^, Xiaoyu Chen ^a, c^, Wang Liao ^a^, Xiuna Jing ^a^, Ming Lei ^a^, Enxiang Tao ^a^, Qiulan Ma ^d^, Xingmei Zhang ^e^, Rui Guo ^b,^ *, Jun Liu ^a, f, g,^ **

^a^ Department of Neurology, Sun Yat-sen Memorial Hospital, Sun Yat-sen University, 107 Yanjiang West Road, Guangzhou, 510120, China

^b^ Key Laboratory of Biomaterials of Guangdong Higher Education Institutes, Department of Biomedical Engineering, Jinan University, Guangzhou 510632, China

^c^ Zhongshan City People’s Hospital, Zhongshan City, Guangdong Province, 528400, China

^d^ Department of Neurology, University of California, Los Angeles, California 90095, USA.

^e^ Applied Immunology and Immunotherapy, Department of Clinical Neuroscience, Karolinska Institute, Center for Molecular Medicine, Karolinska University Hospital at Solna, CMM L8:04, Karolinska Sjukhuset, S-171 76 Stockholm, Sweden

^f^ Laboratory of RNA and Major Diseases of Brain and Heart, Sun Yat-sen Memorial Hospital, Sun Yat-sen University, Guangzhou, 510120, China

^g^ Guangdong Province Key Laboratory of Brain Function and Disease, Zhongshan School of Medicine, Sun Yat-sen University, Guangzhou 510080, China

**^*^** Corresponding author. Key Laboratory of Biomaterials of Guangdong Higher Education Institutes, Department of Biomedical Engineering, Jinan University, Guangzhou 510632, China.

**^**^** Corresponding author. Department of Neurology, Sun Yat-sen Memorial Hospital, Sun Yat-sen University, 107 Yanjiang West Road, Guangzhou, 510120, China.
E-mail addresses for corresponding authors: guorui@jnu.edu.cn (R. Guo), docliujun@hotmail.com (J. Liu).

^1^ These two authors contributed equally to this work.

**Table. S1 M**olecular weight of PLGA-PEG, acrylated PLGA-PEG, PLGA-PEG-B6 and PLGA-PEG-B6/Cur determined by GPC analysis.

| Samples | Mn | Mw | Mw/Mn |
| --- | --- | --- | --- |
| PLGA-PEG | 12000 | 25000 | 2.08 |
| acrylated PLGA-PEG | 12800 | 25600 | 2.00 |
| PLGA-PEG-B6 | 13200 | 26000 | 1.96 |
| PLGA-PEG-B6/Cur | 13600 | 26600 | 1.95 |

**Table. S2** Clotting kinetics parameters of human whole blood mixed with aqueous PLGA-PEG-B6 solutions at different concentrations

| samples | R (min) | K (min) | α (deg) | MA (mm) |
| --- | --- | --- | --- | --- |
| Normal range | 5-10 | 1-3 | 53-72 | 50-70 |
| PBS control | 5.1 | 3.0 | 51.9 | 53.5 |
| 0.01 mg/mL | 5.0 | 3.2 | 52.2 | 53.0 |
| 0.05 mg/mL | 5.0 | 4.8↑ | 40.8↓ | 51.4 |
| 0.1 mg/mL | 6.1 | 5.6↑ | 37.1↓ | 46.2↓ |

**Figure legends:**

**Fig. S1** Particle sizes of PLGA-PEG-B6 and PLGA-PEG-B6/Cur. The mean diameter of PLGA-PEG-B6 particles were less than 100 nm and curcumin encapsulation slightly increase the particle size.

**Fig. S2** Release profiles of curcumin from PLGA-PEG-B6/Cur micelles. The cumulative release of curcumin initially shifted to 29.60% in the first hour, followed by a sustained release for a period of 72 h. Each point represents mean ± SD.

**Fig. S3** The HPLC of B6 peptide

**Fig. S4** The Maldi Toff MS of B6 peptide


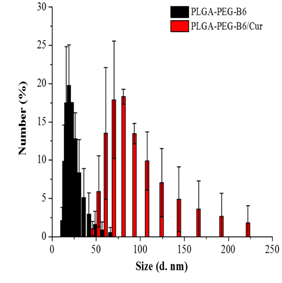


**Fig. S1**.


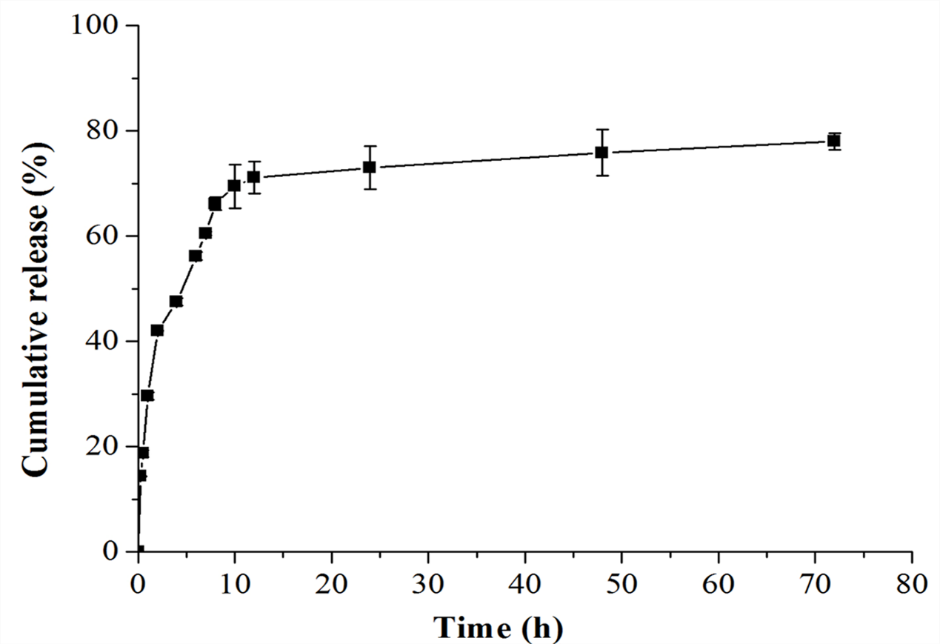


**Fig. S2**.


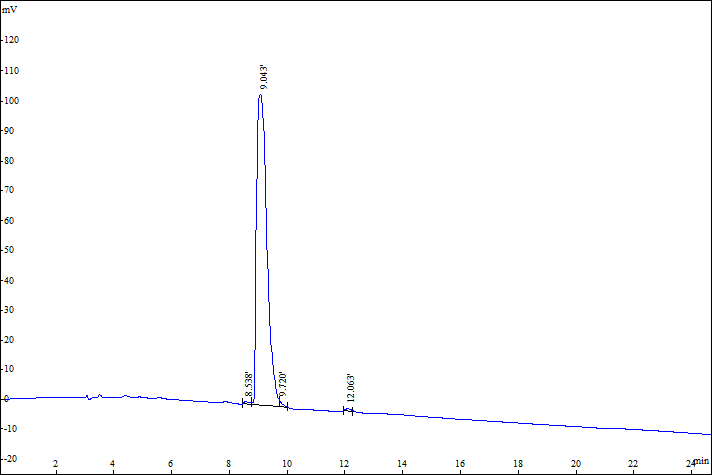


**Fig. S3**

**Fig. S4**
